# Shared acoustic codes underlie emotional communication in music and speech—Evidence from deep transfer learning

**DOI:** 10.1371/journal.pone.0179289

**Published:** 2017-06-28

**Authors:** Eduardo Coutinho, Björn Schuller

**Affiliations:** 1 Department of Music, University of Liverpool, Liverpool, United Kingdom; 2 Department of Computing, Imperial College London, London, United Kingdom; Nanjing Normal University, CHINA

## Abstract

Music and speech exhibit striking similarities in the communication of emotions in the acoustic domain, in such a way that the communication of specific emotions is achieved, at least to a certain extent, by means of shared acoustic patterns. From an Affective Sciences points of view, determining the degree of overlap between both domains is fundamental to understand the shared mechanisms underlying such phenomenon. From a Machine learning perspective, the overlap between acoustic codes for emotional expression in music and speech opens new possibilities to enlarge the amount of data available to develop music and speech emotion recognition systems. In this article, we investigate time-continuous predictions of emotion (Arousal and Valence) in music and speech, and the Transfer Learning between these domains. We establish a comparative framework including intra- (i.e., models trained and tested on the same modality, either music or speech) and cross-domain experiments (i.e., models trained in one modality and tested on the other). In the cross-domain context, we evaluated two strategies—the direct transfer between domains, and the contribution of Transfer Learning techniques (feature-representation-transfer based on Denoising Auto Encoders) for reducing the gap in the feature space distributions. Our results demonstrate an excellent cross-domain generalisation performance with and without feature representation transfer in both directions. In the case of music, cross-domain approaches outperformed intra-domain models for Valence estimation, whereas for Speech intra-domain models achieve the best performance. This is the first demonstration of shared acoustic codes for emotional expression in music and speech in the time-continuous domain.

## Introduction

It is common knowledge that music has the remarkable capacity to stir human emotions and affect our moods in everyday life. Although this is an intuitive fact for most people, understanding the process of emotion induction through music proved to be a major challenge to research in various disciplines. It is not until the past few decades that a consistent body of evidence started unveiling key aspects of such a pervasive phenomenon and shedding new light on the underlying mechanisms supporting the link between music perception and emotion production (see [1, 2]). It is now known that the emotions experienced by music listeners are influenced by a variety of parameters related to listener traits and states, musicians’ performance, and listening and cultural contexts, therefore, rendering the whole process of emotion production listener- and context-dependent (see [2, 3] for detailed accounts). Nonetheless, researchers have also provided important evidence demonstrating that the emotions perceived (i.e., recognised) by listeners in a piece of music seem to depend mostly on particular configurations of acoustic and musical features—it is now well-known that modulations of speed and continuity, accentuation, pitch and range, timbre and dynamics are at the very centre of the communication of emotional meaning (see [4] for an overview), with specific configurations communicating similar emotions universally (e.g., [5–7]).

The fact that the most consistent relationships between musical structure and emotional qualities involve basic variables in human audition rather than complex, music-specific cues, indicates that the way emotions are conveyed through acoustic patterns embedded in music is remarkably similar to the expressive patterns characteristic of speech prosody (the nonverbal aspects of speech that, amongst other things, are crucial in the communication of emotional information). In a meta-analysis that reviews 104 studies of vocal expression and 41 studies of music performance and compared the acoustic characteristics of speech and music associated with particular emotions [8], the authors clearly demonstrate a great degree of overlap between the emotion-specific acoustic patterns of acoustic cues used to express discrete emotions in both domains. Subsequent empirical work, that compared directly both domains, has provided further evidence, both in terms of specific emotions ([9]) as well as in terms of affective dimensions ([10, 11]). In sum, these and other studies provide a strong basis on which to purport the existence of a general mechanism for the expression and recognition of emotions in the acoustic domain (see also [12] for a discussion and account on the possible evolutionary roots of such mechanism).

In this article, we propose to investigate the level of overlap between the acoustic cues to emotion in music and speech. From an Affective Sciences perspective, it is important to explore the level of overlap between the acoustic cues to emotion in music and speech, because it can help explain the reasons why listeners perceive music as expressive of emotion (e.g., [8, 13] by highlighting the underlying mechanisms supporting such phenomenon (e.g. [2, 14]. Furthermore, it can provide support to theories of music origins suggesting that speech and music evolved from a common origin (e.g., [15, 16]). From a Machine Learning perspective, it is advantageous to explore the existence of shared acoustic codes to emotions in both domains since the undifferentiated use of music and speech signals can enlarge the amount of available data which can be used to improve the performance of Speech Emotion Recognition (SER) and Music Emotion Recognition (MER) systems. In particular, the possibility of using speech to develop MER systems is of paramount importance given the fact that there is very little labelled data in this domain. On the other hand, music expresses a wider range of emotional states that are not necessarily present in available speech databases. In that sense, it is also beneficial to develop SER systems by providing a more complete sample of the full emotional spectrum. Additionally, it can lead to the development of hybrid systems, i.e., emotions recognition systems that are applicable to both music and speech signals, which may be particularly important for novel applications in Affective Computing where a holistic understanding of affective signals in the world would be highly beneficial.

### Time-continuous predictions of emotion in music and speech

In [10, 17, 18], it was shown that the prediction of musical affect demands models sensitive to the temporal context of music structural features that continuously adapt the predictions of emotion qualities based on the present and past inputs. This is particularly important in everyday life scenarios where continuous streams of information are always available, and predicting emotion in a time-continuous way is essential to improve interactions between humans and machines. Moreover, it allows to detect both nuanced and evident differences in emotion continuously over time, without determining particular segmentations beforehand. For these reasons, we deal with time-continuous predictions of emotional responses to music and speech.

Only a few works have modelled time-continuous music and speech emotion recognition, and only two have directly compared both domains. In relation to music, the first basic attempt to model time-continuous emotional responses was proposed by [19], who has created multiple linear regression models to predict Arousal and Valence ratings for a set of six music pieces. Nonetheless, such models are not sensitive to the temporal context. In [17], the use of Recurrent Neural Networks (RNN) was introduced to model the same set of music pieces. The authors found that a significant part of the listener’s affective response could be predicted from a small set of six psychoacoustic features—loudness, tempo, texture, mean pitch, pitch variation, and sharpness. This methodology was later successfully applied to a new set of music [18]. Still, a major limitation of these works is the very small set of music pieces resultant from the time-consuming annotation process, which renders doubts about their generality. Such limitations have recently been partially addressed with the introduction of the MediaEval “Emotion in Music” task in 2013 [20], which has led to the creation of a large database of 1000 songs (extended in 2014 to almost 1500 [21]). In the speech domain, [22] applied LSTM-RNNs to the estimation of Arousal and Valence from a subset of natural speech recordings from the SEMAINE database [23] (see also [24]). Recently, the Audio/Visual Emotion Challenges (AVEC) have increasingly focused on time-continuous predictions of emotion from audio (and video) (e.g., [25]).

The first attempt to compare (time-continuous) emotions perceived in music and natural speech was conducted by [10]. The authors applied the same methodology proposed in [17] and [18] to model music and speech separately, and have shown that, an almost identical set of acoustic features allowed to predict the emotions perceived by human listeners in both domains. These variables were loudness, tempo/speech rate, melodic/prosodic contour, spectral centroid, spectral flux, sharpness, and roughness. Unfortunately, like in previous studies, the database size was very limited (8 music pieces and 9 speech samples), and speech and music were modelled separately.

The first direct comparison between both domains (i.e., using the same models and feature sets), albeit in the categorical domain, was presented by [11]. The authors demonstrated that a model trained to classify music or speech instances into a set of discrete emotions using a common set of 200 acoustic features, had a good performance when tested on speech or Music (respectively). Finally, within a time-continuous framework, [26] evaluated whether cross-domain predictions of emotion are a viable option for acoustic emotion recognition. Overall, results indicated a good cross-domain generalisation performance (especially for the model trained on speech and tested on music), but the data used included less than 14 minutes of music and 12 minutes of speech.

In the context of this paper, our aims are two-fold. First, we want to explore the extent to which time-continuous affective-acoustic patterns are generalisable across music and speech, and to predict emotion in music using models trained on emotional speech, and vice-versa. Second, we want to explore the use of knowledge transfer techniques to deal with differences in the feature spaces and distributions of both types of stimuli.

## Background and methods

### Emotion representation

When modelling emotion it is fundamental to define the conceptual model used for its numerical representation. Early work on music emotion recognition focused on classifying music pieces into categorical labels describing specific emotions [27]. The typical lists of emotions (or affect categories) used were the so-called basic emotions (e.g., anger, fear, surprise). Nonetheless, these terms are seldom adequate to describe the emotions present in a music piece and are very dependent on stylistic aspects [28, 29]. In other cases freely chosen tags are used (e.g., [30]), although the quality of this annotation process is questionable (e.g., tags often do not describe emotional states, terms can be highly ambiguous, large number of tags). Moreover, emotions perceived and experienced with music are often ambiguous, mixed and dynamic (e.g., [10, 31]), which demands a more flexible frameworks for emotion representation.

A framework that has proved to be particularly adequate to address these issues are the so-called dimensional models of affects. According to dimensional theorists, the subjective experience of emotion can be depicted by the combination of two or more underlying psychological ‘dimensions’, and therefore that human emotions can be represented as positions in a multidimensional space (the dimensions themselves are usually derived empirically through factorial analysis). A very popular dimensional taxonomy in this domain is the circumplex model of affect proposed by Russell [32], which construes emotions as linear combinations of two independent neurophysiological dimensions—arousal and valence. Arousal describes the level of activation or intensity of the emotions experienced. Valence depicts the hedonic tone of the emotion in a continuum from negative to positive affect. In the context of this work, we adopt Russell’s taxonomy for emotion representation. Apart from its intrinsic benefits, Russell’s taxonomy has the additional advantage of allowing to represent a wide range of emotions that are not specific to music or speech, and that can described by the same variables. This is fundamental because the emotions expressed in speech (triggered by events in everyday life) can be very different from those expressed by music (often of aesthetic nature [2]). Furthermore, dimensional models are nowadays commonly used in SER and MER (e.g., [25, 33])

### Deep long-short term memory recurrent neural networks

From the machine learning point of view, time-continuous predictions of continuous dimensions in a two-dimensional space largely benefit from context-sensitive models. Given their proven ability to model time-continuous emotions in both music (e.g., [34]) and speech (e.g., [35]), we consider the contribution of Long Short-Term Memory RNNs (LSTM-RNNs) [36]. LSTM-RNNs are architecturally similar to the traditional recurrent neural networks (RNN; e.g., [37]) except that, the nonlinear hidden units are replaced by a special kind of memory blocks, which endow the network with the capacity of accessing long-range temporal contexts and predict the outputs based on such information. A single LSTM memory block comprises one (or several) self-connected memory cells and three multiplicative units—input, output and forget gates. These units set up the cells with analogues of write, read and reset operations, and allow LSTM memory cells to store and access information over long sequences (and corresponding periods of time) which permits to overcome the vanishing gradient problem of simple RNNs, whereby the influence of the network inputs on the hidden units (and therefore the outputs) decays or blows up exponentially as the information cycles through the network recurrent connections.

**Fig 1:**
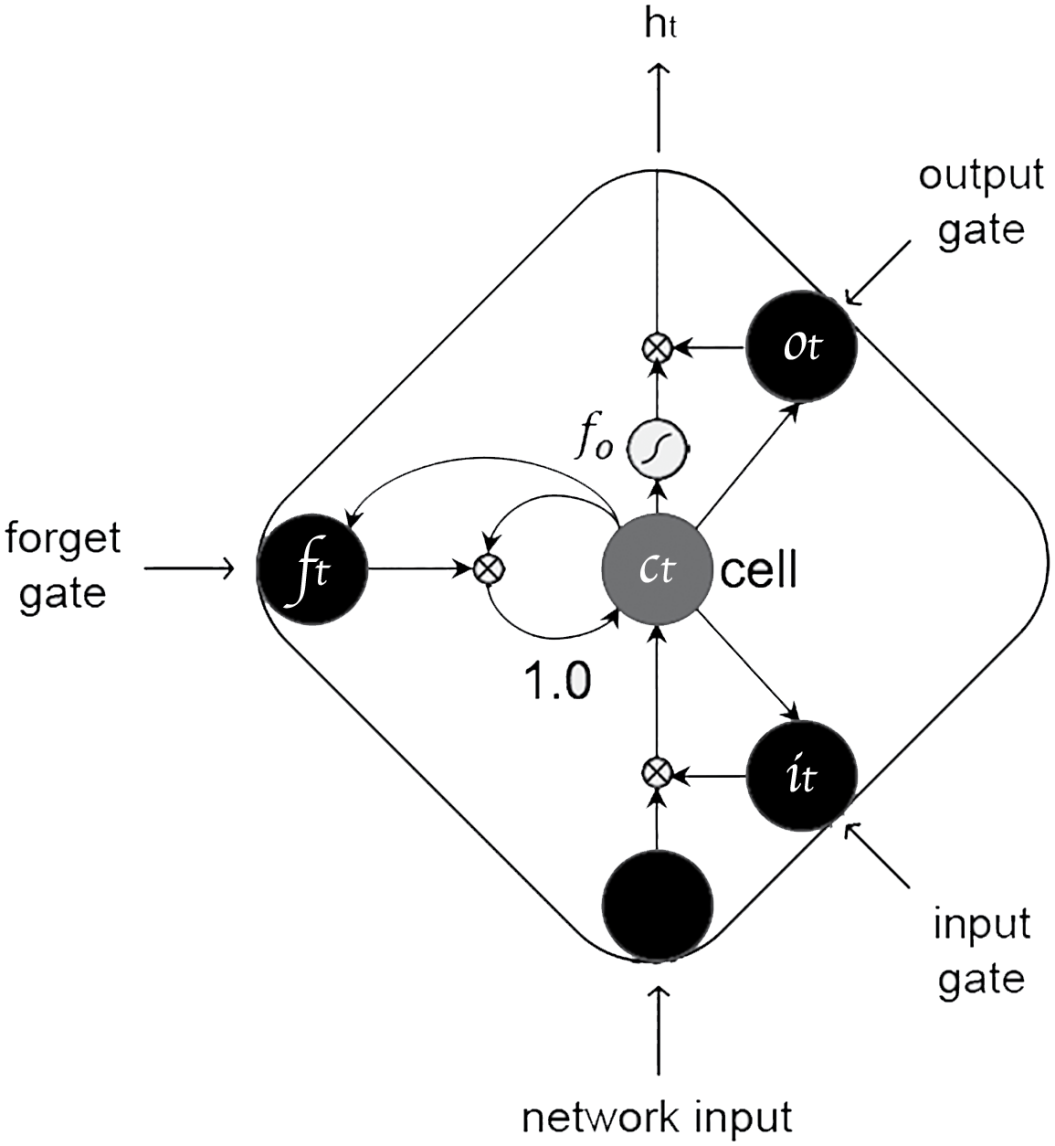
LSTM memory block.

Fig 1 shows a LSTM memory block with one cell. The cell input is initially scaled by the activation function of the input gate. Then, the cell output is computed from the activation of the output gate, plus the memory cell values in the previous time step (controlled by the activation of the forget gates). For a given memory block, with ***W*** as the weight matrix, ***x***_*t*_ the input vector, ***h***_*t*_ the hidden vector, ***b***_*t*_ the hidden bias vector, the activation vector of the input gate ***i***_*t*_ can be expressed as follows:

$$\begin{array}{c} i_{t}=f_{g}\left(W_{xi}x_{t}+W_{hi}h_{t-1}+W_{ci}c_{t-1}+b_{i}\right), \end{array}$$ (1)

where, ***f***_*g*_ is the logistic sigmoid function.

Similarly, the activation of the forget gate ***f***_*t*_ is expressed as:

$$\begin{array}{c} f_{t}=f_{g}\left(W_{xf}x_{t}+W_{hf}h_{t-1}+W_{cf}c_{t-1}+b_{f}\right). \end{array}$$ (2)

The memory cell value ***c***_*t*_ is then the sum of the input vector at time *t* and its own activation in the previous time step:

$$\begin{array}{c} c_{t}=f_{i}\left(W_{xc}x_{t}+W_{hc}h_{t-1}+b_{c}\right)+f_{t}\cdot c_{t-1}, \end{array}$$ (3)

where *f*_*i*_ is the *tanh* activation function.

The output of the memory cell is controlled by the output gate activation:

$$\begin{array}{c} o_{t}=f_{g}\left(W_{xo}x_{t}+W_{ho}h_{t-1}+W_{co}c_{t}+b_{o}\right), \end{array}$$ (4)

Finally, the output of the memory block is:

$$\begin{array}{c} h_{t}=o_{t}\cdot f_{o}\left(c_{t}\right), \end{array}$$ (5)

where *f*_*o*_ is the *tanh* activation function.

Generally, LSTM-RNNs have shown remarkable performance in a variety of Machine Learning tasks, including, handwriting recognition [38], keyword spotting [39], or phoneme classification [40]. Furthermore, as mentioned earlier in this section, they have also been used in the context of time-continuous predictions of emotion in music and speech with considerable success. For more details on LSTM networks the reader is referred to [36].

### Acoustic features for emotion in speech and music

In order to promote the reproducibility of this work, we use a well-developed set for automatic recognition of paralinguistic phenomena—the official feature set of the 2013 INTERSPEECH Computational Paralinguistics Challenge (*ComParE*). This feature set is already a standard in SER (e.g., [41, 42]) and MER (e.g., [21, 33]). The *ComParE* feature set comprises 65 of low-level audio descriptors (LLDs; see Table 1) and their first order derivates (*Δ* LLDs; a total of 130 features), which cover a broad set of descriptors from the fields of speech processing, Music Information Retrieval, and general sound analysis. For the computation of all LLDs, we used overlapping windows with a step size of 10 ms. LLDs related to voice were computed using 60 ms long time frames and Gaussian windows (*σ* = 0.4), whereas LLDs related to all other features were calculated using 25 ms long time frames and Hamming window functions (leading to 17% and 40% overlaps, respectively). In order to adapt the sampling rate of the feature set to that of the annotations, we also computed the mean and standard deviation functionals of each feature over 2 s time windows with 50% overlap (step size of 1.0 s). This resulted in a total of 260 features extracted at a rate of 1 Hz. The computation of these features was done using the open-source feature extractor openSMILE ([43]). For full details on the *ComParE* feature set please refer to [11].

**Table 1:** ComParE feature set: List of 65 energy-, spectral- and voicing-related low-level descriptors (LLD).

**Energy related LLDs (4)**	**Group**
Sum of auditory spectrum (loudness)	prosodic
Sum of RASTA-style filtered auditory spectrum	prosodic
RMS Energy	prosodic
**Spectral LLDs (55)**	**Group**
RASTA-style auditory spectrum, bands 1–26 (0–8 kHz)	spectral
MFCC 1–14	cepstral
Spectral energy 250–650 Hz, 1 k–4 kHz	spectral
Spectral Roll off point 0.25, 0.50, 0.75, 0.90	spectral
Spectral Flux, Centroid, Entropy, Slope	spectral
Psychoacoustic Sharpness, Harmonicity	spectral
Spectral Variance, Skewness, Kurtosis	spectral
Zero-Crossing Rate	prosodic
**Voicing related LLDs (6)**	**Group**
*F*_0_ (SHS & Viterbi smoothing)	prosodic
Prob. of voice	sound quality
log. HNR, Jitter (local, delta), Shimmer (local)	sound quality

### Transfer learning with denoising auto-encoders

The concept of Transfer Learning (TL) is inspired by the fact that humans can use previously acquired knowledge in one domain to solve new problems, faster and efficiently, in new domains. In the realm of Machine Learning, TL has been proposed to deal with the problematic situation of having training and test samples derived from different feature spaces and data distributions. In this scenario, most statistical models need to be retrained with new data that matches the new distributions, which is an expensive process and often unrealistic. As an example, in the SER field, a common limitation of models trained on a specific speech corpus is the tendency to under-perform with recordings from other sources. This can be due to various reasons, but often is related to the characteristics of the speakers in each corpus, the type of emotions being conveyed, the level of portrayal vs spontaneity, the recording conditions, among others (see [44]). In point of fact, TL has recently started to be applied in this area with evident success ([45]).

In this work, we explore the existence of shared acoustic codes communicating emotions in music and speech, and evaluate the extent to which TL can benefit the generalisation of emotion recognition across domains. Considering that we are using a common taxonomy to represent emotions in both domains, we have a typical *transductive transfer learning* (*tTL*) setting [46]—the source (*T*_*S*_) and target (*T*_*T*_) tasks are the same (*T*_*S*_ = *T*_*T*_; regression of emotion dimensions), but the source (*D*_*S*_) and target (*D*_*T*_) domains are different (*D*_*S*_ ≠ *D*_*T*_). Specifically, given that in our case the source and target feature spaces are the same, but the marginal probability distributions of the inputs for each domain are different, our task is related to domain adaptation [47]. Given *D*_*S*_ and *D*_*T*_ (with respective learning tasks *T*_*S*_ and *T*_*T*_), *tTL* aims to improve the learning of the target predictive function *f*_*T*_(.) in *D*_*T*_ using the knowledge in *D*_*S*_ and *T*_*S*_ (*D*_*S*_ ≠ *D*_*T*_ and *T*_*S*_ = *T*_*T*_).

For the transfer of knowledge related to emotion decoding from music (or speech) to speech (or music), our aim is to find adequate feature representations that maximise domain convergence and the regression model error. In particular, in our task only a relatively small amount of labelled data is available in the source domain *D*_*S*_ and no labelled data exists on the target domain *D*_*T*_. Typically, under these settings, transferring knowledge of features representations (aka feature-representation-transfer) is achieved through unsupervised learning frameworks [46]. Several techniques have been proposed for unsupervised feature-representation-transfer (e.g., see [46] for a detailed overview). In this paper, we focus on representation learning [48] in order to learn transformations of the data in the source and target feature domains that ease the extraction of useful representations for inputting to the emotion regression models. In particular, we focus on directly learning a parametric map from the features to their representations which, unlike learned representations based on latent variables, does not require that the posterior distribution is known and allows extracting stable deterministic numerical feature values [48]. One such class of methods is the regularised version of the auto-encoder (AE) framework [49] that forces the AE to develop more general internal feature representations (as insensitive as possible with respect to changes in input). Popular techniques implementing this method are Sparse Auto-Encoders [50] and Denoising Auto-Encoders (DAE) [51], with the latter often associated with better outcomes [52]. DAEs are trained to reconstruct a clean, ‘repaired’ input from its corrupted version [51], that is, to denoise corrupted versions of their inputs. In so doing, the DAE must capture the structure of the input distribution in order to reduce the effect of the corruption process, and as a consequence learn (useful) higher level representations. This method has been shown to be efficient in a wide range of tasks (see [52]), including, recently, in SER (e.g., [45]).

## Databases

In our TL test-bed, we established a (realistic) scenario in which a large pool of unlabelled data is pervasively available in both domains (e.g., speech and music from online sources), but only a relatively small amount of labelled data is available in the source domain *D*_*S*_ and none in the target domain *D*_*T*_. In our experiments we further establish two sub-scenarios, whereby music is the source domain and speech the target domain, and vice-versa. The next subsections describe the four databases used (two of music and two of speech instances). Our choices for the labelled databases were driven by the quality and reliability of the annotations in order to have a robust set of data for our Machine Learning experiments, as well as their availability which can facilitate reproducibility of this work.

### Speech

#### DAE pre-training: Semaine database

The SEMAINE corpus [53] was developed specifically to address the task of achieving emotion-rich interactions, and it is adequate for this task as it comprises a wide range of emotional speech. It includes video and speech recordings of spontaneous interactions between human and emotionally stereotyped ‘characters’ (Prudence, who is even-tempered and sensible; Poppy, who is happy and outgoing; Spike, who is angry and confrontational, and Obadiah, who is sad and depressive). Audio was recorded at 48 kHz with 24 bits per sample. In our experiments, we use a subset of this database (called *Solid-SAL*), which is freely available for scientific research purposes (see http://semaine-db.eu), and it was used as the official database in the First International Audio/Visual Emotion Challenge (AVEC 2011) [54]. In this subset of SEMAINE, the characters are role-played by human operators. Table 2 shows the details of *Solid-SAL* database.

**Table 2:** Overview of the speech databases used in this paper (f: female).

Database	SEMAINE	RECOLA
Number of recordings	95	23
Number of speakers	28 (14 f)	23 (12 f)
Total duration (h:m:s)	7:30:29	1:55:00
Total number of frames (1 s long)	27 029	6 900

#### Regression task: RECOLA database

The RECOLA database [55] (the official database of AVEC 2015, the 5th International Audio/Visual Emotion Challenge and Workshop [56]) consists of 9.5 hours of multimodal recordings (audio, video, and peripheral physiological activity) of spontaneous dyadic interactions between French adults whom were performing a remote collaborative task. Initially, each participant was asked to rank a number of items according to their importance for the survival of a group of crew members in a deserted and hostile area after a plain crash. Then, they had to discuss their ratings with another peer and reach a consensus on how to survive in the proposed disaster scenario (mean duration of the interactions was circa 15 minutes).

In this paper, we use the RECOLA-Audio module which consists of the audio recordings of each participant in the dyadic phase of the task. In particular, we use the non-segmented high-quality audio signals (WAV format, 44.1kHz, 16bits), obtained through unidirectional headset microphones, of the first five minutes of each interaction. These sections contain the most emotionally expressive moment of the interactions, and include annotations of perceived emotions by six French speakers. Annotations consist of time-continuous ratings of the level of Arousal and Valence dimensions of emotion perceived by each rater while seeing and listening the audio-visual recordings of each participant task.

The details of the sound files used are shown in Table 2. Given that not all participants consented to the release of their recordings, and the authors only released part of the data from the annotated sound files, the total number of instances is 23. The time frame length used is this work is 1s given that no changes in time-continuous annotations are expected at lower rates [19].

It should be noted that the RECOLA database contains speech in the French language, whereas SEMAINE is an English database. In that respect it should noted that we are focusing on the non-linguistic aspects of speech (prosody) and that cross-cultural research has demonstrated that emotions conveyed by the human voice (and by music as well) can be communicated accurately across cultures and that convincing evidence points to similar prosodic codes underlying this phenomenon (e.g., [57, 58]. This is apparent, for instance, in our capacity to understand how other feel even if they speak an unfamiliar language.

### Music

#### DAE pre-training: MediaEval 2014 (ME14)

The MediaEval “Emotion in Music” task is dedicated to the estimation of Arousal and Valence scores continuously in time and value for song excerpts from the Free Music Archive. The whole corpus (development and test sets for the 2014 challenge) includes 1 744 songs belonging to 11 musical styles—Soul, Blues, Electronic, Rock, Classical, Hip-Hop, International, Folk, Jazz, Country, and Pop (maximum of five songs per artist). The numerical details of the *ME14* corpus are shown in Table 3.

**Table 3:** Overview of the Mediaeval “Emotion in Music” task corpus (*ME14*) and database derived from previous Music Psychology empirical studies (*MP*).

Database	ME14	MP
Number of pieces	1000	20
Number of genres	11	8
Total duration (h:m:s)	8:19:59	1:28:50
Total number of frames (1 s long)	30 000	5 749

#### Regression task: Compilation of data from Music Psychology studies (MP)

The music database compiled for this study consists of emotionally diverse full music pieces from a variety of musical styles (“Classical” and contemporary Western Art, Baroque, Bossa Nova, Rock, Pop, Heavy Metal, and Film Music). Each piece was administered in the context of controlled laboratory experiments in which the emotional character of each piece was evaluated time-continuously by 35 to 52 participants using a computer mouse to control a cursor on the screen to indicate the continuous level of Arousal and Valence perceived at each moment [10, 17, 18, 59]. In what follows, we describe each study. The numerical information pertaining to the various datasets is summarised in Table 4.

**Table 4:** Music database: Collection of music pieces used in five different Music Psychology studies and employed in our work for the Arousal and Valence regression tasks.

Dataset	*MP*_*DB*_1__	*MP*_*DB*_2__	*MP*_*DB*_3__	*MP*_*DB*_4__
Number of pieces	6	9	8	7
Number of genres	1	1	1	7
Total duration (m:s)	18:38	23:40	13:11	33:21
Number of time frames (1s long)	1 118	1 420	791	2 001

*MP*_*DB*_1__ This subset of our database consists of the data reported in [60], and gently made available by the author. This dataset includes six full (or long excerpts) music pieces ranging from 151 s to 315 s in length (only classical music). Each piece was annotated by 35 participants (14 females). The time series correspondents to each music piece were collected at 1 Hz. The golden standard for each piece was computed by averaging the individual time series across all raters.

*MP*_*DB*_2__ The dataset by [18] includes 9 full pieces (43s to 240s long) of classical music (romantic repertoire) annotated by 39 subjects (19 females). Values were recorded every time the mouse was moved with a precision of 1 ms. The resultant timeseries were then resampled (moving average) to a synchronous rate of 1 Hz. The golden standard for each piece was computed by averaging the individual time series across all raters.

*MP*_*DB*_3__ The music in the third study [10] consists of 8 pieces of film music (84 s to 130 s long) taken from the late 20^th^ century Hollywood film repertoire. Emotion ratings were given by 52 participants (26 females). The annotation procedure, data processing, and golden standard calculations were identical to *MP*_*DB*_2__.

*MP*_*DB*_4__ The music used in [59] includes seven music pieces (127 s to 502 s in length) of heterogeneous styles (e.g., Rock, Pop, Heavy Metal, Classical). Each music piece was annotated by 38 participants (29 females) using an identical methodology to *MP*_*DB*_2__ and *M*_*DB*_3__. Data processing and golden standard calculations were also identical.

## Experimental setup, procedure and measures

In the next subsections we describe our experimental procedure. The contents are organised in two subsections. In the first, we attempt to use DAEs for feature-representation-transfer with the aim of improving the cross-modal generalisation. The DAEs are trained on the large, unlabelled music and speech databases (*SEMAINE* and *ME*14). In the second part, we conduct the regression experiments on the target tasks (predicting Arousal and Valence) using a three-part comparative framework. First, we created a baseline to allow establishing a reference to compare against the performance of TL. The baseline consists of intra domain models, i.e., models developed and tested only on music or speech. Second, given that we use the same feature set for both domains, we establish a basic cross-domain setup whereby a model is trained with data from one modality and tested with data from another. Third, we attempt to use DAEs for feature-representation-transfer with the aim of improving the cross-modal generalisation. In all cases we conducted bidirectional experiments, that is, transfer learning from music to and from speech.

### Pre-training of the first layer

The first step was to pre-train the first layer of the network using a DAE framework. The DAE takes as input time-continuous feature vectors of acoustic descriptors, and attempt to reproduce these time series at the output units from corrupted version of the inputs. In this paper, we inject Gaussian noise with zero mean and variable standard deviation (*σ*; see details below) at the input layer (only during training) in order to generate a corrupted version of inputs during training (see [61] for other methods of corruption). In all tests and trials the DAEs were trained on the *ME*14 (music) and *SEMAINE* (speech) databases in an unsupervised fashion. Also, we computed 10 trials (repetitions) in each test condition, each with randomised initial weights in the range [-0.1, 0.1] in order to obtain a more robust performance estimation. Training was stopped once there was no improvement after 20 consecutive learning iterations, or a maximum of 300 iterations was reached. The architecture implemented for each DAE consisted of an input layer composed of 260 units with sigmoidal activation functions, one hidden layer composed of a variable number of LSTM blocks (#*HB*_*layer*_1__), and one output layer composed of 260 units also with sigmoidal activation functions.

In order to understand how the transfer learning process is affected by the size of the hidden layer (the dimensionality of the feature vector to be transferred) and the *σ* of the noise injected in the input layer (which will impact the generalisability of the feature vector), we trained different DAEs with a variable number of hidden layer sizes (#*HB*_*layer*_1__ ∈ [50, 75, 100, 150, 200]) and different noise parameters. Each combination of #*HB*_*layer*_1__ and *σ* will later be used tested in the regression experiments (as mentioned, for each parameter set combination (#*HB*_*layer*_1__, *σ* and *lr*_*dae*_), we computed 10 independent trials of the models with randomised initial weights in the range [-0.1, 0.1]).

The only parameter optimised in the pre-training phase was the learning rate (*lr*_*dae*_). This optimisation was done in two steps. First, for each value of #*HB*_*layer*_1__, we trained the model with different learning rates (*lr*_*dae*_ ∈ [1 × 10^−7^, 1 × 10^−6^, 5 × 10^−6^]; *momentum* = 0.9 and *σ* = 0.1 for all tests) (again this process was repeated 10 times for each #*HB*_*layer*_1__/*lr*_*dae*_ pair). The optimal *lr*_*dae*_ for each #*HB*_*layer*_1__ was determined by selecting the lowest average reconstruction error (computed using the *rmse*) over the 10 trials. Second, for each #*HB*_*layer*_1__ (using the optimised *lr*_*dae*_ for each layer size), we varied the characteristics of the noise injected at the inputs (*σ* ∈ [0.1, 0.2, 0.3, 0.4, 0.5, 0.6]) (also repeated 10 times for each #*HB*_*layer*_1__/*σ* combination). As a result, we created 10 different DAEs (each with an optimised learning rate) that correspond to 30 different initialisation of the first layer to be used in the regression tests (each with 10 repeated trials).

Note that the size of the hidden layers is smaller than the number of inputs features given that we expect redundancy amongst features. By setting the hidden layer to be smaller than the input layer, our goal is to implicitly reduce the dimensionality of the feature space by eliminating redundancy and improving generalisation performance.

### Models training on the target task

We used a multi-task learning framework for the joint learning of Arousal and Valence time-continuous values. In all tests, the network’s basic architecture consisted of 260 inputs (the number of acoustic features), two hidden layers (with LSTM memory blocks) of variable size, and two output units with linear activation functions (corresponding to Arousal and Valence outputs). In all cases, mirroring the pre-training phase, we computed ten trials (repetitions) in each test condition, each with randomised initial weights in the range [-0.1, 0.1]. We conducted three separate blocks of experiments—intra-domain (*ID*), cross-domain (*CD*), and cross-domain with TL (*CD*_*TL*_):

- *ID* experiments correspond to the traditional approaches—separate models were created for each domain (music and speech) using the extracted acoustic features. During training, for each domain, we used a leave-one-out nested cross-validation schema (*N*_*music*_ = 33 and *N*_*speech*_ = 23). In each fold, *N* − 2 instances are used for training (training set), one is used for parameter optimisation (validation set), and another instance is left out for testing (test set). The best parameter set is determined as the average performance over the left-out instances of each fold. Once the best model was found, its performance to unseen data was determined as the average performance over the test instances of each fold.
- *CD* experiments focused on developing the model with data from one domain (music or speech) and directly tested on the other domain (speech or music, respectively). In the model development and optimisation phase, we used a leave-one-out cross-validation strategy. The performance for each condition was estimated as the average performance over the left-out instances of each training fold. The optimised model was then tested on cross-domain data (music if trained on speech, and vice-versa).
- *CD*_*TL*_ experiments were also focused on creating the models with data from the source domain (music or speech) and test them on the target domain (speech or music, respectively). Again, we used a leave-one-out cross-validation strategy in the development phase using the source domain database, and the model performance was estimated on the database of the target domain. The key difference between *CD*_*TL*_ and *CD* is that the first layer was not randomly initialised. Instead, we initialised the first layer with the weights and biases matrix of the DAEs described in the previous sections. Tests were conducted with each (*lr*_*dae*_, #*HB*_*layer*_1__) pair.

#### Architecture and hyper-parameter optimisation

Each sequence (music or speech instance) was presented randomly to the model during training, and both the input and output data were standardised to the mean zero and unit variance (using the parameters of the correspondent training sets in each cross-validation fold).

For all models, we optimised the number of hidden LSTM blocks in each layer using a learning rate of 1*e*^−6^ (no Gaussian noise was applied to the inputs, and a momentum of 0.9 was used). The size of the first hidden layer was varied between 50 and 200 neurons (#*HB*_*layer*_1__ ∈ [50, 75, 100, 150, 200]). As noted, in the case of the *CD*_*TL*_ experiments, we initialised the weight and biases matrices of the first layer with the parameters of the previously trained DAEs (note that the sizes of the first hidden layer are the same). In practice, this was done by removing the output layer of the previously trained DAEs, and adding an extra hidden layer and a new output layer (composed by two units: Arousal and Valence). These weights were kept fixed while training the models on the target task. Given that we trained 6 DAEs with the same number of hidden units (due to the various *σ*_*DAE*_ used), unlike the *ID* and *CD* experiments, we conducted 6 tests (instead of one) per hidden layer in order to test the impact of the *σ*_*DAE*_ value used in the pre-training phase.

After determining the #*HB*_*layer*_1__ size (or #*HB*_*layer*_1__/*σ*_*DAE*_ pair in the case of the *CD*_*TL*_ experiments) yielding the best performance on the target tasks, we optimised the size of the second hidden layer on the target task (#*HB*_*layer*_2__ ∈ [2, 4, 6, 8, 10]). Then, using the optimal architectures (#*HB*_*layer*_1__ and #*HB*_*layer*_2__ sizes) we optimised the learning rate (*LR*_*model*_ ∈ [1 × 10^−7^, 1 × 10^−6^, 5 × 10^−6^]). Finally, we injected Gaussian noise with different standard deviations (*σ*_*model*_; mean was 0) in the input layer to regularise the training process (*σ*_*model*_ ∈ [0.1, 0.2, 0.3, 0.4, 0.5, 0.6]). In addition to the input noise, we used an early stopping strategy to avoid over-fitting the training data. The training process was halted after 20 iterations without improvement to the performance of the validation set, and a maximum of 500 iterations of the learning algorithm was allowed.

## Performance measures

We use three measures to quantify the models’ performance: an index of precision—the root mean squared error (*rmse*), a measure of similarity—Pearson’s linear correlation coefficient (*r*), and a measure that combines both—the Concordance Correlation Coefficient (*ccc*; [62]). Given that the trade-off between precision and similarity of the temporal paths is fundamental for an accurate estimation of unfolding of the emotional expression (particularly in the case of music since in speech fast changes in emotional tone are less likely to occur in most circumstances), we optimised the model using the *ccc* cost function. We report *rmse* and *r* for clarity, and because it allows to better interpret the results given that *ccc* is a compound measure. The *rmse* is measured in the same units as the data, and should therefore be interpreted carefully. Given that model outputs (as well as the golden standard) vary between -1 and 1, an informative way is to consider a normalised version of the *rmse*, which can be achieved by diving the original *rmse* values by the range of observed values (in our case 1 − (−1) = 2. This value is an intuitive measure of the magnitude of the error. The interpretation of *r* is straightforward—larger values indicate a better linear correlation between model outputs and target values. Our analysis will be guided by the interpretation of *ccc*, *r* and *rmse*. *ccc* provides a global indication of the models’ performances in terms of precision (also depicted by *rmse*) and similarity (measured by *r*) of the affective trajectories for each piece. In order to determine whether the different conditions yielded results that are significantly different from each other, we conducted statistical comparisons between the performances in each experiment using Student’s *t*-tests with Bonferroni corrections for multiple comparisons (three tests, therefore the p-values were multiplied by three).

## Results

### DAE pre-training

In Table 5, we show the statistics on the reconstruction errors of the DAEs with different number of hidden blocks and noise variability (*σ*) applied to the inputs. These results were obtained with the optimised learning rate for each hidden layer size (as described in the previous section; values also indicated in the table: *LR*_*dae*_). As it can be observed, the DAEs could reproduce very well the input features for all parameters tested. The optimised learning rate tended to increase for higher values of *σ*, as well as the *rmse*. *r* tended to decrease for increasing *σ*. The best reproductions of the inputs were achieved for smaller sizes of the hidden layer.

**Table 5:** DAE performance for different hidden layer sizes, and various variances of the Gaussian noise *σ* applied to the inputs during training. For each combination of *σ* and #*LSTMblocks*, we indicate the mean-squared error *rmse* and Person’s linear correlation coefficient *r* (*rmse* / *r* for each cell).

*LR*_*dae*_	*σ*	#*LSTMblocks*				
		50	75	100	150	200
5 × 10^−7^	0.1	27 / 0.808	21 / 0.880	16 / 0.926	12 / 0.950	9 / 0.968
1 × 10^−6^	0.2	28 / 0.806	21 / 0.881	16 / 0.928	13 / 0.950	10 / 0.969
1 × 10^−6^	0.3	28 / 0.804	21 / 0.881	17 / 0.921	14 / 0.942	13 / 0.955
1 × 10^−6^	0.4	28 / 0.799	22 / 0.872	18 / 0.909	16 / 0.930	14 / 0.943
1 × 10^−6^	0.5	29 / 0.791	22 / 0.863	19 / 0.899	17 / 0.916	16 / 0.928
5 × 10^−6^	0.6	29 / 0.785	23 / 0.851	20 / 0.887	19 / 0.902	18 / 0.912

### Regression task

In Table 6, we summarise the complete set of results obtained for the MER (left) and SER (right) regression tasks in the three experimental conditions: intra-domain (*M*2*M* and *S*2*S*), cross-domain (*M*2*S* and *S*2*M*), and cross-domain with TL (*M*2*S*_*TL*_ and *S*2*M*_*TL*_). *D*_*S*_2*D*_*T*_ indicates the direction of knowledge transfer—*D*_*S*_ is the source domain (*M*usic or *S*peech), and *D*_*T*_ is the target domain (*S*peech or *M*usic). The performance figures shown correspond to the average of each measure across the five best trials in each experimental condition, and are shown separately for Arousal and Valence for better interpretability. In the table, we also indicate the values of the hyper-parameters optimised in the development phase.

**Table 6:** Results obtained for the test sets of the various experimental conditions (Music on the left side, and Speech on the right). The performance of the various approaches is quantified using the root-mean-squared-error (*rmse*), Pearson’s linear correlation coefficient *r*, and the concordance correlation coefficient *ccc*. For each experiment, the performance measures were averaged across the five best trials. *ID*: intra-domain (*M*2*M* and *S*2*S*, baseline models trained and tested on the same database); *CD*: cross-domain (*S*2*M* and *M*2*S*); *CD*_*TL*_, cross-domain transfer learning (*S*2*M* and *M*2*S*); *D*_*S*_ ⇒ *D*_*T*_ (where *D*_*S*_ is the source domain, and *D*_*T*_ the target domain).

		MUSIC			SPEECH		
		Intra-Domain	Cross-Domain		Intra-Domain	Cross-Domain	
		*M*2*M*	*S*2*M*	*S*2*M*_*TL*_	*S*2*S*	*M*2*S*	*M*2*S*_*TL*_
First layer size		150	200	200	150	200	150
Second layer size		10	10	10	10	8	10
Learning rate		5 × 10^−6^	5 × 10^−6^	5 × 10^−6^	5 × 10^−6^	5 × 10^−6^	5 × 10^−6^
Gaussian noise *σ*		0.5	0.5	0.4	0.4	0.1	0.1
Arousal	*rmse*	0.194	0.223	0.223	0.105	0.142	0.141
	*r*	0.517	0.524	0.542	0.821	0.693	0.719
	*ccc*	**0.317**	**0.276**	**0.269**	**0.749**	0.545	0.567
Valence	*rmse*	0.265	0.260	0.265	0.109	0.143	0.151
	*r*	0.175	0.256	0.218	0.448	0.228	0.246
	*ccc*	0.090	**0.133**	0.117	**0.332**	0.164	0.181

Before describing the results pertaining to the focus of this article (the comparisons between the experimental conditions), it is important to make some considerations about the quality of the predictions. As it can be observed in Table 6, results show very good fits to the Arousal data for both Music and Speech experiments, and moderate fits to Valence. Indeed, in relation to Arousal, *r* > 0.5 in all experiments which suggest an excellent fit to the data. Furthermore, all *rmse*’s are smaller than 0.223, which, considering that the range of the outputs is [-1, 1], corresponds to a maximum normalised *rmse* of approximately 11% (0.223/2), and therefore also an excellent precision. The results concerning Valence are clearly less positive, which is not surprising given the fact that Valence is harder to be perceived by people and automatically estimated (see, for instance, [10, 21]). This is particularly evident in terms of similarity (*r* can be as low as 0, 175), but not so accentuated in terms of precision (the worst normalised *rmse* was 13%, which is similar to Arousal). Generally, these results indicate a good fit the data in both domains, and confirm our expectations in terms of modelling emotional responses to music and speech using acoustic descriptors (in this case the ComPaRe 2013 feature set).

#### Music as the target domain

We focus now on the statistical analysis of the experiments where Music was used as the target domain, i.e., *M*2*M*, *S*2*M* and *S*2*M*_*TL*_).

In relation to Arousal, results show that all conditions achieved statistically the same performance in terms of *ccc* and *r* (*p* > 0.05 in all cases). In terms of precision, the performance of the cross-domain models was slightly worse than the model only trained on music (*t*(*M*, *S*2*M*) = −3.96, *p* = 0.004, *t*(*M*, *S*2*M*_*TL*_) = −4.12, *p* = 0.003).

Regarding Valence, the two cross-modal models performed generally better than the baseline model. Indeed, the statistical analysis of the *ccc* values showed that the performances of *S*2*M* and *S*2*M*_*TL*_ were significantly higher than the baseline model (*t*(*M*, *S*2*M*) = −3.84, *p* = 0.005 and *t*(*M*, *S*2*M*_*TL*_) = −2.86, *p* = 0.021). Furthermore, the *S*2*M* model performed better than *S*2*M*_*TL*_ (*t*(*S*2*M*, *S*2*M*_*TL*_) = 2.36, *p* = 0.046), and therefore yielded the best performance. There were no differences in terms of precision, but the *S*2*M* lead to statistically better results than the *M*2*M* model in terms of *r* (*t*(*M*, *S*2*M*) = −3.51, *p* = 0.008).

**Fig 2:**
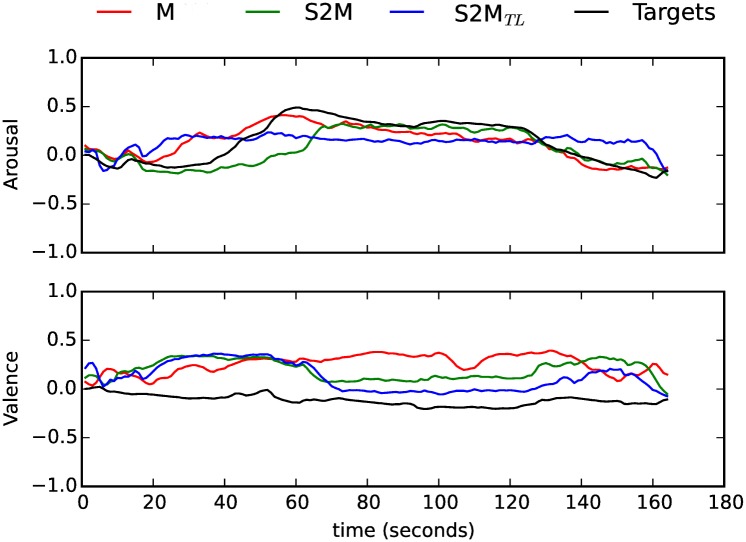
Time-continuous Arousal and Valence predictions by the intra- (*M*) and cross-domain (*S*2*M*, *S*2*M*_*TL*_) models for *M*inority Report, Main Theme (taken from *M*_*DB*_3__, piece 6 in [10]). The golden standard is also shown (‘Targets’).

**Fig 3:**
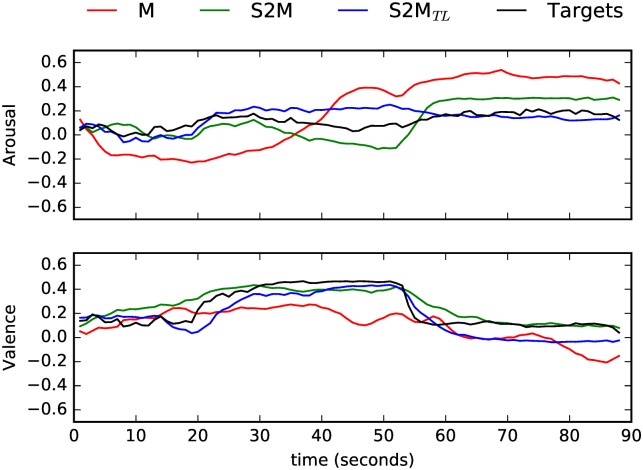
Time-continuous Arousal and Valence predictions by the intra- (*M*) and cross-domain (*S*2*M*, *S*2*M*_*TL*_) models for a representative music piece: *T*he Searchers, Suite (taken from*M*_*DB*_3__, piece 8 in [10]). The golden standard is also shown (‘Targets’).

In sum, the models trained on speech (with or without the pre-trained layer) delivered statistically the same performance of the baseline model trained and tested on music for Arousal, and the *S*2*M* model delivered the best results for Valence. In Figs 2 and 3 we show the Arousal and Valence predictions for two music pieces (used here as examples; they do not necessarily represent the best or worst instances). The Arousal and Valence values shown were obtained from the best models in each condition (*M*, *S*2*M* and *S*2*M*_*TL*_). We also represent the respective golden standards.

#### Speech as the target domain

**Fig 4:**
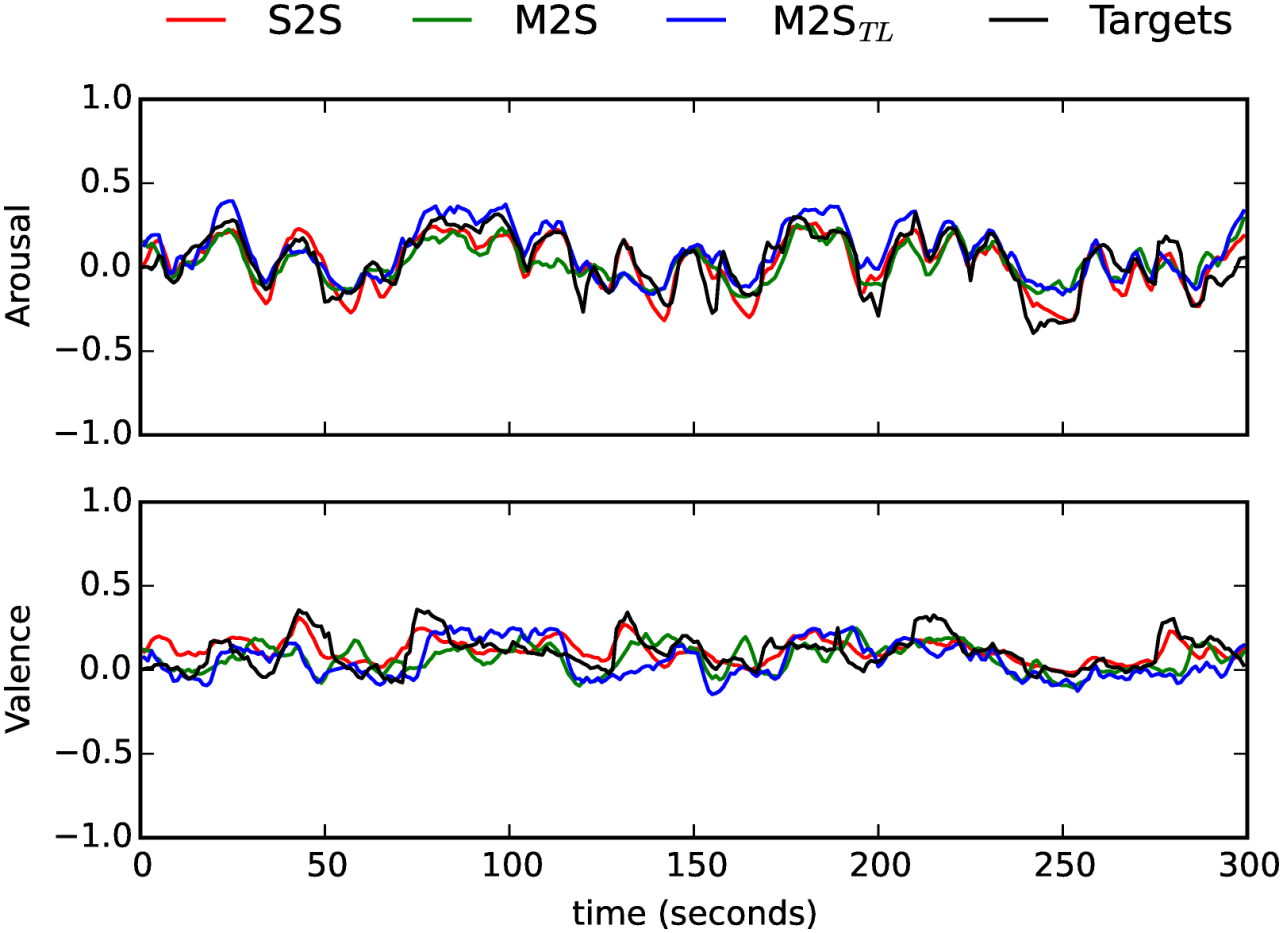
Time-continuous Arousal and Valence predictions by the intra- (*S*) and cross-domain (*M*2*S*, *M*2*S*_*TL*_) models for a RECOLA speech sample (male speaker; instance id: ‘Recola_P26_GRP13_2_HQ’). The golden standard is also shown (’Targets’).

**Fig 5:**
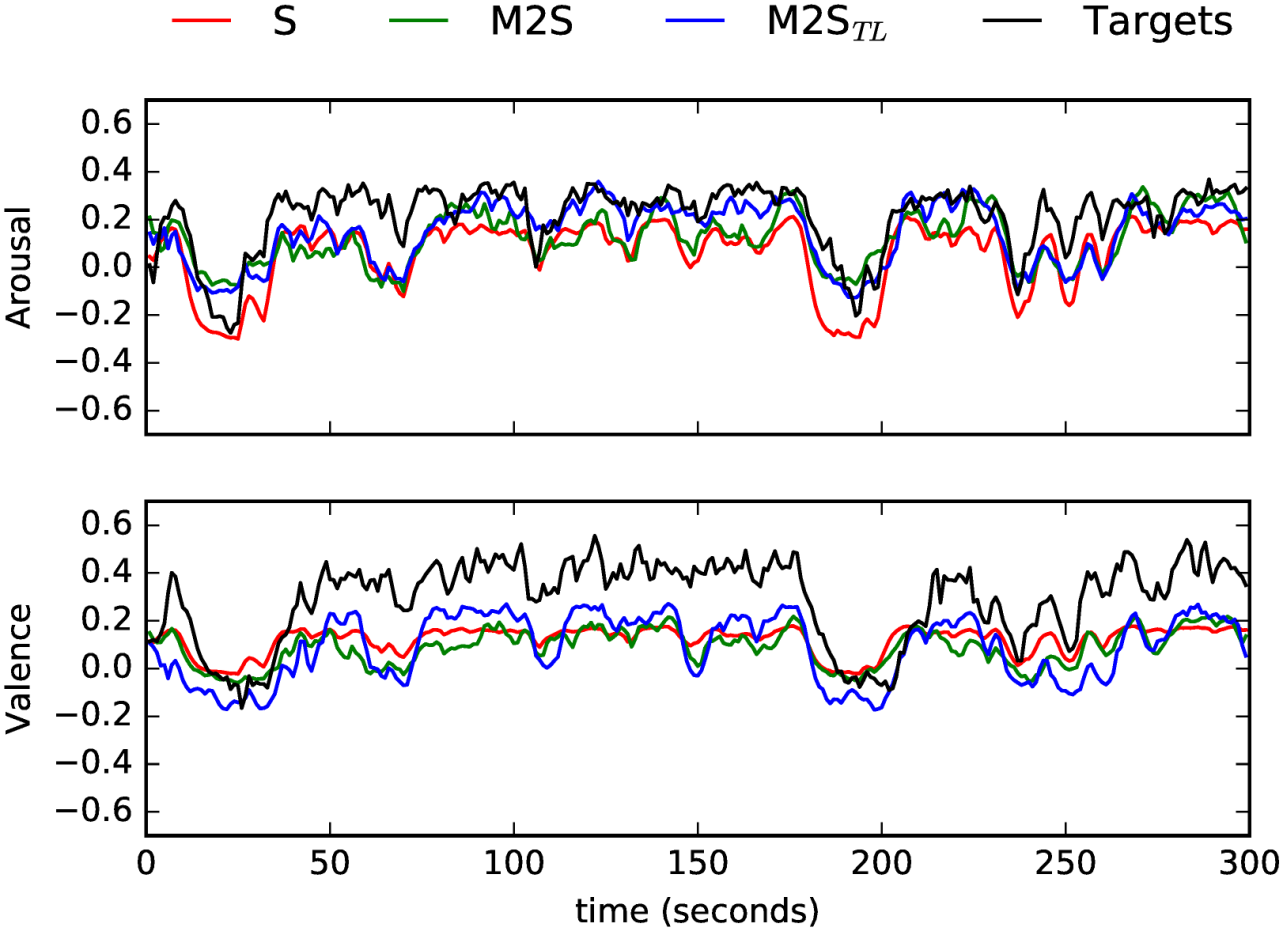
Time-continuous Arousal and Valence predictions by the intra- (*S*) and cross-domain (*M*2*S*, *M*2*S*_*TL*_) models for a RECOLA speech sample (male speaker; instance id: ‘Recola_P56_GRP28_2_HQ’). The golden standard is also shown (’Targets’).

We turn now to the experiments where Speech was the target domain. Looking first at Arousal, the baseline model yielded higher *ccc* than the cross-domain models (*t*(*S*, *M*2*S*) = 31.2, *p* < 0.001, *t*(*S*, *M*2*S*_*TL*_) = 14.7, *p* < 0.001). This was also the case both in terms of *r* (*t*(*S*, *M*2*S*) = 7.21, *p* < 0.001; *t*(*S*, *M*2*S*_*TL*_) = 9.59, *p* < 0.001), and *rmse* (*t*(*S*, *M*2*S*) = −9.54, *p* < 0.001; *t*(*S*, *M*2*S*_*TL*_) = −10.7, *p* < 0.001). There were no statistically significant differences between both cross-domain models (*p* > 0.05). The results pertaining Valence are identical. The intra-domain model *S* performed globally better than the cross-domain models (*t*(*S*, *M*2*S*) = 24.2, *p* < 0.001, *t*(*S*, *M*2*S*_*TL*_) = 15.9, *p* < 0.001), and there were no significant differences between the cross-domain models *M*2*S* and *M*2*S*_*TL*_. The differences in the global performance are related to a decreased performance in terms of *r* for both cross-domain models (*t*(*S*, *M*2*S*) = 15.1, *p* < 0.001, *t*(*S*, *M*2*S*_*TL*_) = 16.3, *p* < 0.001), and a decrease in terms of precision (*t*(*S*, *M*2*S*) = −2.43, *p* = 0.041; *t*(*S*, *M*2*S*_*TL*_) = −5.28, *p* = 0.001). In Figs 4 and 5, we show the predicted Arousal and Valence by the best models in each condition (*S*, *M*2*S* and *M*2*S*_*TL*_)) against the respective golden standards for two speech samples (once more the two instances selected do not necessarily represent the best or worst instances—they are used for illustrative purposes only).

## Discussion and conclusions

In this article, we focused on time-continuous predictions of emotion in music and speech, and investigating the overlap between these two tasks. Indeed, as discussed at the outset, given the tight relationships between the acoustic codes for emotional expression in music and speech, it is plausible to assume that, the communication of emotion in both domains may be achieved by means of shared acoustic patterns. In this context, we proposed to transfer knowledge from one domain to the other with the aims of exploring the extent of the overlap between domains, and testing whether it is possible to enlarge the amount of data available to develop MER and SER systems by combining both domains. In our experiments, we established a comparative framework including intra- (i.e., models trained and tested on the same modality, either music or speech) and cross-domain experiments (i.e., models trained in one modality and tested on the other). The intra-domain models were used as a baseline. In the cross-domain context, we evaluated two strategies—the direct transfer of knowledge between domains, and the contribution of Transfer Learning techniques, namely feature-representation-transfer based on Denoising Auto Encoders.

There are various relevant conclusions and observations deriving from our results and analyses. Starting with overall performances per domain, we found that the speech regression tasks delivered a much better performance compared to the music task for both affective dimensions (Arousal and Valence). Furthermore, as expected from most previous work on Affective Sciences and Affective Computing, Arousal predictions were more accurate than those of Valence for both domains. The fact that we obtained a better performance for the speech compared to music may simply be the consequence of the datasets used, nonetheless there are at least two other possible interpretations. First, predicting emotion in music may be a more challenging task (especially in relation to Valence). This is plausible to assume for various reasons, including the fact that emotion recognition in music is more stereotyped and listener dependant (e.g., [2]), and that valence can be ambivalent in music (e.g., [63]). Furthermore, emotions expressed in the voice have a functional (biological) role, and are meant to communicate specific emotional meanings, something that does not necessarily happen in the case of music [2]. Second, the music model may be under-performing because the feature set used was initially developed for speech-related tasks, and, albeit commonly and effectively used for music tasks, may be lacking important features for music emotion recognition. In point of fact, in [34] it was shown that, the addition of only a few features related to duration, dissonance (roughness), and sharpness to the feature set used in this paper can significantly improve intra-domain music emotion recognition.

We turn now to our considerations concerned with the central focus of this paper—the comparison between intra- and cross-domain experiments. In the of case of Music as the target domain, we found that the Arousal predictions in all three conditions (*M*2*M*, *S*2*M* and *S*2*M*_*TL*_) were similar, although the cross-domain models were slightly worse in terms of precision. The cross-modal models (*S*2*M* and *S*2*M*_*TL*_) performed significantly better than the baseline model (*M*2*M*) when predicting Valence, and there were no significant differences between both. In relation to Speech as the target domain, for both Arousal and Valence predictions, the intra-domain models (*S*2*S*) performed significantly better than the cross-domain models (*M*2*S* and *M*2*S*_*TL*_), which achieved statistically the same performance. These results evidence two important aspects relating to the transfer of knowledge between domains:

1. Knowledge transfer (with or without domain adaptation) from speech to music was more successful than in the opposite direction. Indeed, emotions in music could be predicted with models trained on speech or music equally, whereas the speech model trained on speech data outperformed those trained on music.
2. Knowledge transfer approaches with and without domain adaptation lead to the statistically same results for both Music and Speech.

**Fig 6:**
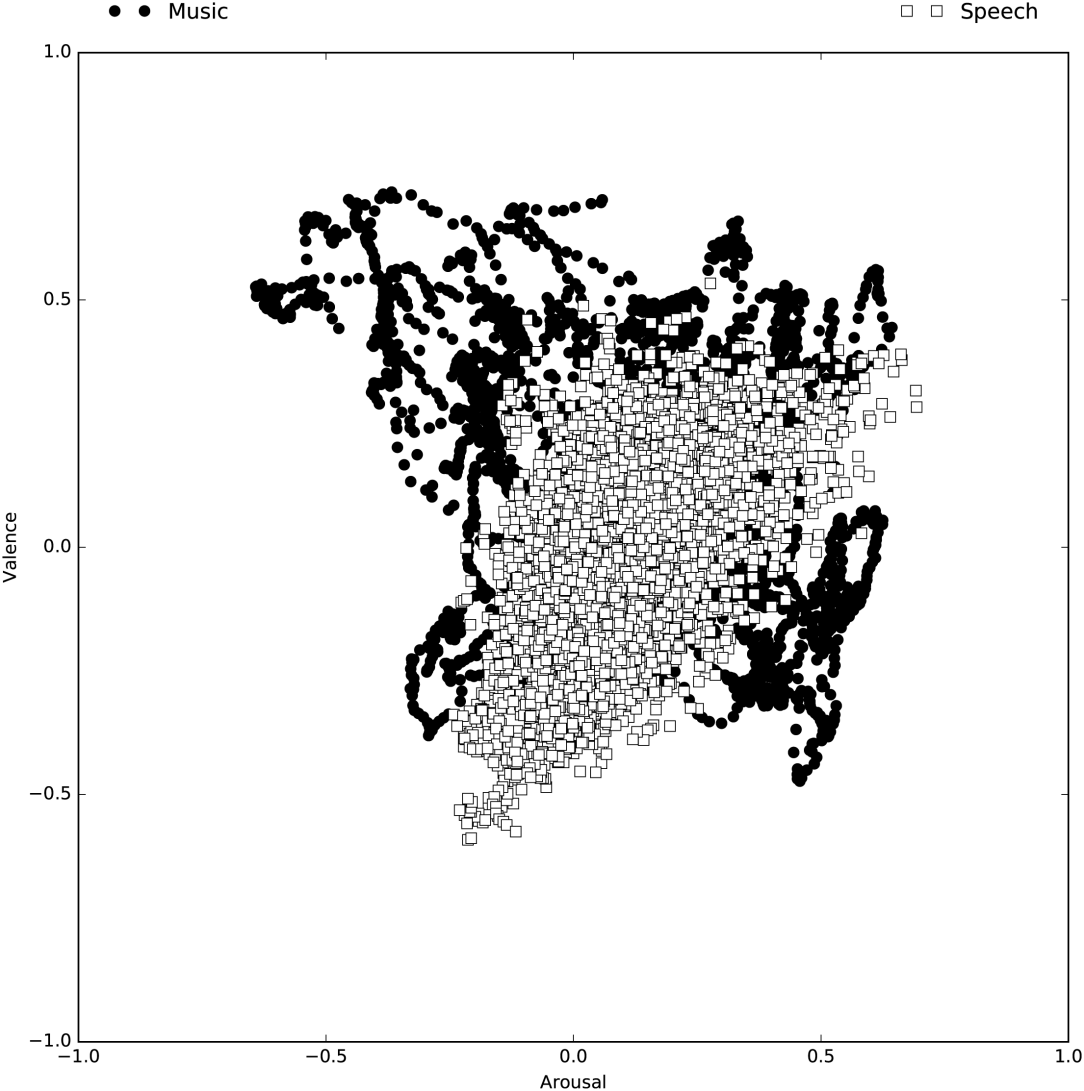
Distribution of the target annotations of the databases used in the regression tasks. Dots and squares (music and speech, respectively) represent the Arousal and Valence values at each time step for all instances used in the regression experiments (time is not represented).

In relation to (1), our results can simply reflect the nature of the datasets, and particularly the distributions of targets for each database. This is evident in Fig 6, which shows that music and speech emotions ratings in the databases used are only partially overlapping—there are zones in the bi-dimensional space formed by Arousal and Valence that are covered by the music database, but not by the speech instances. The most clear examples are the top-left (high Arousal and negative Valence) and bottom-right (low Arousal and positive Valence) quadrants, but it is generally visible that the emotions perceived in music are more varied. Another possible justification for the knowledge transfer asymmetry is the fact that music makes use of acoustic patterns to convey emotions that are not used in speech, and therefore such knowledge is not transferable. This is plausible considering the much wider range of sounds (compared to speech) that is possible to produce with music instruments (and nowadays computers), and their qualities and organisation in time. In this context, there may be a wider range of emotion conveying mechanisms at work, derived from a wider range of acoustic sources available in music, and possibilities to combine sounds in meaningful ways (including emotional). This is coherent with contemporary theories of music evolution, such as the *musilanguage* model [12], which posits that, music has specialised in the communication of affective meaning (whereas language specialised in the communication of referential meaning). Furthermore, there is evidence of brain specialisations for music, which are related, among other aspects, to the encoding of pitch along musical scales and the ascribing of a regular beat to incoming acoustics signals [64]. Such brain networks may also encode important information about music, and particularly patterns related to the recognition of emotion. In that case, as mentioned above, it is also possible that the standard features used lack relevant information specific to music.

Regarding (2), our findings reveal two important pieces of evidence: a) the standard cross-domain approach led to excellent cross-domain generalisations; b) knowledge transfer by feature-representation-learning did not lead to significant improvements over the standard cross-domain approach. One the one hand these findings indicate that the cross-domain models perform well even without adaptation of the feature space, reinforcing the ideas of a close coupling between both domain in terms of acoustic codes for emotional communication. On the other hand, considering that we have used deep neural networks in both cross-domain experiments (with the same number of hidden layers), it may indicate that, the pre-training process did not extract additional, relevant statistical information from the features spaces of the source and target domains. In this case, larger, more varied unlabelled databases may be needed to sample a sufficient amount of information from the features in both domains. Also, deeper architectures may favour the extraction of relevant representations at different structural levels (see, for instance, [65] for stacked DAEs with sparse rectifier units approach), and the fine-tuning of the pre-trained layer during the regression task may be also advantageous.

In sum, our results show an evident and excellent cross-domain generalisation of time-continuous estimations of emotional Arousal and Valence in music and speech even without the support of Transfer Learning (in this case feature-representation-transfer). This is a clear demonstration that there is a substantial overlap between the acoustic codes for emotional expression in music and speech, which can help explain the power of music to communicate emotions to its listeners by confirming the existence of shared acoustic codes for emotional communication. These findings are also beneficial for computer scientists interested in automatic estimation of emotion due to the fact that data from either domain (music or speech) can be used to develop models emotion recognition models. This is particularly relevant in contexts where the amount of data in either domain is scarce, which is clearly the case of music, but also in contexts where the whole emotional spectrum is not represented by a particular dataset and can be complement by data from another domain (the emotions typically experienced with music are a different subset from those most commonly experiences in everyday life circumstances; see for instance [28, 29]. Finally, we would like to highlight some limitations of this work. The first one pertains to the datasets used. Time continuous labelled databases of music and speech are scarce, and the datasets used here are limited in that they cannot cover the whole emotional spectrum. As mentioned, this may have limited the transfer of knowledge from music to speech. Second, we have only tested a particular Transfer Learning technique and did not perform adaptation on the regression targets. In future work, we plan to explore alternative Transfer Learning techniques (e.g. functional transfer) to further evaluate whether it can benefit the knowledge transfer between features spaces, as well as attempt to deal with the mismatches between the distributions of the regression outputs. Third, we worked with a feature set that was optimised for Speech Emotion Recognition, even though it is commonly (and successfully) used for Music Emotion Recognition tasks. In future work, we intend to conduct more detailed analysis on the characteristics of feature space and enlarge it with new cross- and intra-domain features.

## References

[1] Juslin PN. From everyday emotions to aesthetic emotions: towards a unified theory of musical emotions. Physics of life reviews. 2013;10(3):235–66. 10.1016/j.plrev.2013.05.008 23769678

[2] Scherer KR, Coutinho E. How music creates emotion: a multifactorial process approach In: Cochrane T, Fantini B, Scherer KR, editors. The Emotional Power of Music: Multidisciplinary perspectives on musical arousal, expression, and social control. 1st ed Oxford, UK: Oxford University Press; 2013 p. 121–146.

[3] Scherer KR, Zentner M. Emotional effects of music: production rules In: Juslin PN, Sloboda JA, editors. Music and emotion: theory and research. Oxford/New York: Oxford University Press; 2001 p. 361–392.

[4] Gabrielsson A, Lindström E. The role of structure in the musical expression of emotions In: Juslin PN, Sloboda J, editors. Handbook of music and emotion: Theory, research, applications. Oxford: Oxford University Press; 2010 p. 367–400.

[5] Balkwill LL, Thompson WF. A cross-cultural investigation of the perception of emotion in music: psychophysical and cultural cues. Music Perception. 1999;17(1):43–64. 10.2307/40285811

[6] Balkwill LL, Thompson WF, Matsunaga R. Recognition of emotion in Japanese, Western, and Hindustani music by Japanese listeners. Japanese Psychological Research. 2004;46(Special Issue: Cognition and emotion in music):337–349. 10.1111/j.1468-5584.2004.00265.x

[7] Fritz T, Jentschke S, Gosselin N, Friederici AD, Koelsch S. Report Universal Recognition of Three Basic Emotions in Music. Current Biology. 2009;19(7):573–576. 10.1016/j.cub.2009.02.058 19303300

[8] Juslin PN, Laukka P. Communication of emotions in vocal expression and music performance: different channels, same code? Psychological bulletin. 2003;129(5):770–814. 10.1037/0033-2909.129.5.770 12956543

[9] Ilie G, Thompson WF. Experiential and Cognitive Changes Following Seven Minutes Exposure to Music and Speech. Music Perception: An Interdisciplinary Journal. 2011;28(3):247–264. 10.1525/mp.2011.28.3.247

[10] Coutinho E, Dibben NJ. Psychoacoustic cues to emotion in speech prosody and music. Cognition & Emotion. 2012;26:658–684. 10.1080/02699931.2012.732559 23057507

[11] Weninger F, Eyben F, Schuller BW, Mortillaro M, Scherer KR. On the Acoustics of Emotion in Audio: What Speech, Music, and Sound have in Common. Frontiers in Psychology. 2013;4(May):1–12. 23750144 10.3389/fpsyg.2013.00292 PMC3664314

[12] Brown S. The “musilanguage” model of music evolution In: Wallin NL, Merker B, Brown S, editors. The Origins of Music. Cambridge, MA: MIT Press; 2000 p. 271–300.

[13] Kivy P. A failure of aesthetic emotivism. Philosophical studies. 1980;38(4):351–365. 10.1007/BF00419335

[14] Juslin PN, Västfjäll D. Emotional responses to music: The need to consider underlying mechanisms. Behavioral and brain sciences. 2008;31(05):559–575. 10.1017/S0140525X08005293 18826699

[15] Brown. The “Musilanguage” Model of Music Evolution In: Wallin N, Merker B, Brown S, editors. The Origins of Music. Cambridge, MA: The MIT Press; 1999 p. 271–301.

[16] Scherer KR. Expression of emotion in voice and music. Journal of voice. 1995;9(3):235–248. 10.1016/S0892-1997(05)80231-0 8541967

[17] Coutinho E, Cangelosi A. The Use of Spatio-Temporal Connectionist Models in Psychological Studies of Musical Emotions. Music Perception. 2009;27(1):1–15. 10.1525/mp.2009.27.1.1

[18] Coutinho E, Cangelosi A. Musical Emotions: Predicting Second-by-Second Subjective Feelings of Emotion From Low-Level Psychoacoustic Features and Physiological Measurements. Emotion. 2011;11(4):921–937. 10.1037/a0024700 21859207

[19] Schubert E. Modeling perceived emotion with continuous musical features. Music perception. 2004;21(4):561–585. 10.1525/mp.2004.21.4.561

[20] Soleymani M, Caro MN, Schmidt EM, Sha CY, Yang YH. 1000 Songs for Emotional Analysis of Music. In: Proceedings of the 2Nd ACM International Workshop on Crowdsourcing for Multimedia. CrowdMM’13. New York, NY, USA: ACM; 2013. p. 1–6. Available from: http://doi.acm.org/10.1145/2506364.2506365

[21] Aljanaki A, Yang YH, Soleymani M. Emotion in music task at mediaeval 2014. In: Working Notes Proceedings of the MediaEval 2014 Workshop. vol. 1263. CEUR-WS.org; 2014.

[22] Wöllmer M, Eyben F, Reiter S, Schuller B, Cox C, Douglas-Cowie E, et al. Abandoning Emotion Classes—Towards Continuous Emotion Recognition with Modelling of Long-Range Dependencies. In: Proceedings INTERSPEECH 2008, 9th Annual Conference of the International Speech Communication Association, incorporating 12th Australasian International Conference on Speech Science and Technology, SST 2008. ISCA/ASSTA. Brisbane, Australia: ISCA; 2008. p. 597–600.

[23] Douglas-Cowie E, Cowie R, Sneddon I, Cox C, Lowry O, McRorie M, et al. The HUMAINE Database: Addressing the Collection and Annotation of Naturalistic and Induced Emotional Data. In: Paiva ACR, Prada R, Picard RW, editors. Affective Computing and Intelligent Interaction. vol. 4738 of Lecture Notes in Computer Science. Berlin-Heidelberg, Germany: Springer; 2007. p. 488–500. Available from: 10.1007/978-3-540-74889-2_43

[24] Wöllmer M, Schuller B, Eyben F, Rigoll G. Combining long short-term memory and dynamic bayesian networks for incremental emotion-sensitive artificial listening. Selected Topics in Signal Processing, IEEE Journal of. 2010;4(5):867–881. 10.1109/JSTSP.2010.2057200

[25] Ringeval F, Schuller B, Valstar M, Cowie R, Pantic M. AVEC 2015: The 5th international audio/visual emotion challenge and workshop. In: Proceedings of the 23rd ACM international conference on Multimedia. ACM; 2015. p. 1335–1336.

[26] Coutinho E, Deng J, Schuller B. Transfer Learning Emotion Manifestation Across Music and Speech. In: Proceedings 2014 International Joint Conference on Neural Networks (IJCNN) as part of the IEEE World Congress on Computational Intelligence (IEEE WCCI). IEEE. Beijing, China: IEEE; 2014. p. 3592–3598.

[27] Feng Y, Zhuang Y, Pan Y. Popular Music Retrieval by Detecting Mood. In: Proceedings of the 26th Annual International ACM SIGIR Conference on Research and Development in Informaion Retrieval. SIGIR’03. New York, NY, USA: ACM; 2003. p. 375–376. Available from: http://doi.acm.org/10.1145/860435.860508

[28] Zentner M, Scherer KR, Grandjean D. Emotions evoked by the sound of music: characterization, classification, and measurement. Emotion. 2008;8(4):494–521. 10.1037/1528-3542.8.4.494 18729581

[29] Coutinho E, Scherer KR. Introducing the GEneva Music-Induced Affect Checklist (GEMIAC): A Brief Instrument for the Rapid Assessment of Musically Induced Emotions. Music Perception. 2017;34:371–386. 10.1525/mp.2017.34.4.371

[30] Lin YC, Yang YH, Chen HH. Exploiting Online Music Tags for Music Emotion Classification. ACM Trans Multimedia Comput Commun Appl. 2011;7S(1):26:1–26:16. 10.1145/2037676.2037683

[31] Hunter PG, Schellenberg EG, Schimmack U. Mixed affective responses to music with conflicting cues. Cognition & Emotion. 2008;22(2):327–352. 10.1080/02699930701438145

[32] Russell JA. A circumplex model of affect. Journal of personality and social psychology. 1980;39(6):1161 10.1037/h0077714 10948981

[33] Aljanaki A, Yang YH, Soleymani M. Emotion in music task at mediaeval 2015. In: Working Notes Proceedings of the MediaEval 2015 Workshop. vol. 1436. CEUR-WS.org; 2015.

[34] Coutinho E, Weninger F, Schuller B, Scherer KR. The Munich LSTM-RNN Approach to the MediaEval 2014 “Emotion in Music” Task. In: Working Notes Proceedings of the MediaEval 2014 Workshop. vol. 1263. Barcelona, Spain: CEUR-WS.org; 2014. p. 5–6.

[35] Ringeval F, Eyben F, Kroupi E, Yuce A, Thiran JP, Ebrahimi T, et al Prediction of asynchronous dimensional emotion ratings from audiovisual and physiological data. Pattern Recognition Letters. 2014;66:22–30. 10.1016/j.patrec.2014.11.007

[36] Gers FA, Schmidhuber J, Cummins F. Learning to forget: Continual prediction with LSTM. Neural Computation. 2000;12(10):2451–2471. 10.1162/089976600300015015 11032042

[37] Elman JL. Finding structure in time. Cognitive science. 1990;14(2):179–211. 10.1207/s15516709cog1402_1

[38] Graves A. Supervised Sequence Labelling with Recurrent Neural Networks. vol. 385 of Studies in Computational Intelligence. Berlin-Heidelberg, Germany: Springer-Verlag; 2012.

[39] Wöllmer M, Schuller B, Batliner A, Steidl S, Seppi D. Tandem decoding of children’s speech for keyword detection in a child-robot interaction scenario. ACM Transactions on Speech and Language Processing (TSLP). 2011;7(4):12.

[40] Graves A, Schmidhuber J. Framewise phoneme classification with bidirectional LSTM and other neural network architectures. Neural Networks. 2005;18(5):602–610. 10.1016/j.neunet.2005.06.042 16112549

[41] Schuller B, Steidl S, Batliner A, Vinciarelli A, Scherer K, Ringeval F, et al. The INTERSPEECH 2013 Computational Paralinguistics Challenge: Social Signals, Conflict, Emotion, Autism. In: Proceedings INTERSPEECH 2013, 14th Annual Conference of the International Speech Communication Association. ISCA. Lyon, France: ISCA; 2013. p. 148–152.

[42] Schuller B, Steidl S, Batliner A, Epps J, Eyben F, Ringeval F, et al. The INTERSPEECH 2014 Computational Paralinguistics Challenge: Cognitive & Physical Load. In: Proceedings INTERSPEECH 2014, 15th Annual Conference of the International Speech Communication Association. ISCA. Singapore, Singapore: ISCA; 2014. p. 427–431.

[43] Eyben F, Weninger F, Groß F, Schuller B. Recent Developments in openSMILE, the Munich Open-Source Multimedia Feature Extractor. In: Proceedings of the 21st ACM International Conference on Multimedia, MM 2013. Barcelona, Spain: ACM; 2013. p. 835–838.

[44] Schuller B, Vlasenko B, Eyben F, Wollmer M, Stuhlsatz A, Wendemuth A, et al Cross-corpus acoustic emotion recognition: Variances and strategies. Affective Computing, IEEE Transactions on. 2010;1(2):119–131. 10.1109/T-AFFC.2010.8

[45] Deng J, Zhang Z, Marchi E, Schuller B. Sparse autoencoder-based feature transfer learning for speech emotion recognition In: Proc. ACII. HUMAINE Association. Geneva, Switzerland: IEEE; 2013 p. 511–516.

[46] Pan SJ, Yang Q. A Survey on Transfer Learning. IEEE Transactions on Knowledge and Data Engineering. 2010;22(10):1345–1359. 10.1109/TKDE.2009.191

[47] Arnold A, Nallapati R, Cohen WW. A Comparative Study of Methods for Transductive Transfer Learning. In: Data Mining Workshops, 2007. ICDM Workshops 2007. Seventh IEEE International Conference on. IEEE; 2007. p. 77–82.

[48] Bengio Y, Courville A, Vincent P. Representation learning: A review and new perspectives. Pattern Analysis and Machine Intelligence, IEEE Transactions on. 2013;35(8):1798–1828. 10.1109/TPAMI.2013.50 23787338

[49] Bourlard H, Kamp Y. Auto-association by multilayer perceptrons and singular value decomposition. Biological cybernetics. 1988;59(4-5):291–294. 10.1007/BF00332918 3196773

[50] Ranzato M, Poultney C, Chopra S, Cun YL. Efficient Learning of Sparse Representations with an Energy-Based Model In: Schölkopf B, Platt JC, Hoffman T, editors. Advances in Neural Information Processing Systems 19. MIT Press; 2007 p. 1137–1144.

[51] Vincent P, Larochelle H, Bengio Y, Manzagol PA. Extracting and Composing Robust Features with Denoising Autoencoders. In: Proceedings of the 25th International Conference on Machine Learning. ICML’08. New York, NY, USA: ACM; 2008. p. 1096–1103. Available from: http://doi.acm.org/10.1145/1390156.1390294

[52] Vincent P, Larochelle H, Lajoie I, Bengio Y, Manzagol PA. Stacked denoising autoencoders: Learning useful representations in a deep network with a local denoising criterion. The Journal of Machine Learning Research. 2010;11:3371–3408.

[53] McKeown G, Valstar M, Cowie R, Pantic M, Schroder M. The SEMAINE Database: Annotated Multimodal Records of Emotionally Colored Conversations between a Person and a Limited Agent. IEEE Transactions on Affective Computing. 2012;3:5–17. 10.1109/T-AFFC.2011.20

[54] Schuller B, Valstar M, Cowie R, Pantic M, editors. Proceedings of the First International Audio/Visual Emotion Challenge and Workshop, AVEC 2011. vol. 6975, Part II of Lecture Notes on Computer Science (LNCS). HUMAINE Association. Memphis, TN: Springer; 2011.

[55] Ringeval F, Sonderegger A, Sauer J, Lalanne D. Introducing the RECOLA multimodal corpus of remote collaborative and affective interactions. In: Automatic Face and Gesture Recognition (FG), 2013 10th IEEE International Conference and Workshops on. Shanghai, China: IEEE; 2013. p. 1–8.

[56] Ringeval F, Schuller B, Valstar M, Cowie R, Pantic M. AVEC 2015—The 5th International Audio/Visual Emotion Challenge and Workshop. In: Proceedings of the 23rd ACM International Conference on Multimedia, MM 2015. ACM. Brisbane, Australia: ACM; 2015. p. 1335–1336.

[57] Ilie G, Thompson WF. A Comparison of Acoustic Cues in Music and Speech for Three Dimensions of Affect. Music Perception. 2006;23(4):319–330. 10.1525/mp.2006.23.4.319

[58] Thompson WF. Decoding speech prosody in five languages. Semiotica. 2006;158(1/4):407–424.

[59] Grewe O, Nagel F, Kopiez R, Altenmüller E. Emotions over time: synchronicity and development of subjective, physiological, and facial affective reactions to music. Emotion (Washington, DC). 2007;7(4):774–88. 10.1037/1528-3542.7.4.774 18039047

[60] Korhonen M. Modeling Continuous Emotional Appraisals of Music Using System Identification; 2004. Available from: http://hdl.handle.net/10012/879

[61] Alain G, Bengio Y. What regularized auto-encoders learn from the data-generating distribution. Journal of Machine Learning Research. 2014;15(1):3563–3593.

[62] Lin L. A concordance correlation coefficient to evaluate reproducibility. Biometrics. 1989;45(1):255–268. 10.2307/2532051 2720055

[63] Kawakami A, Furukawa K, Katahira K, Okanoya K. Sad music induces pleasant emotion. Frontiers in Psychology. 2013;4(311):1–15. 23785342 10.3389/fpsyg.2013.00311 PMC3682130

[64] Peretz I. Brain specialization for music. New evidence from congenital amusia. Annals of the New York Academy of Sciences. 2001;930:153–65. 10.1111/j.1749-6632.2001.tb05731.x 11458826

[65] Glorot X, Bordes A, Bengio Y. Domain adaptation for large-scale sentiment classification: A deep learning approach. In: Proceedings of the 28th International Conference on Machine Learning. Bellevue, WA, USA: International Machine Learning Society; 2011. p. 513–520.

