# Correction: Shared acoustic codes underlie emotional communication in music and speech—Evidence from deep transfer learning

**DOI:** 10.1371/journal.pone.0191754

**Published:** 2018-01-19

**Authors:** Eduardo Coutinho, Björn Schuller

The link provided in the Data Availability statement is incorrect. The correct link is: https://doi.org/10.5281/zenodo.345944.
